# A Path-Planning Approach Based on Potential and Dynamic Q-Learning for Mobile Robots in Unknown Environment

**DOI:** 10.1155/2022/2540546

**Published:** 2022-06-02

**Authors:** Bing Hao, He Du, Jianshuo Zhao, Jiamin Zhang, Qi Wang

**Affiliations:** College of Computer and Control Engineering, Qiqihar University, Qiqihar, China

## Abstract

The path-planning approach plays an important role in determining how long the mobile robots can travel. To solve the path-planning problem of mobile robots in an unknown environment, a potential and dynamic Q-learning (PDQL) approach is proposed, which combines Q-learning with the artificial potential field and dynamic reward function to generate a feasible path. The proposed algorithm has a significant improvement in computing time and convergence speed compared to its classical counterpart. Experiments undertaken on simulated maps confirm that the PDQL when used for the path-planning problem of mobile robots in an unknown environment outperforms the state-of-the-art algorithms with respect to two metrics: path length and turning angle. The simulation results show the effectiveness and practicality of the proposal for mobile robot path planning.

## 1. Introduction

With the development of artificial intelligence, mobile robotics are widely used in various fields: medical robots that assist patients or people with disabilities [1, 2]; teaching robots that provide flexible platforms for educational research [3]; swarm intelligent robots capable of military applications [4, 5]; and cleaning robots that serve people's productive lives [6]. These applications are all dependent on the path planning of mobile robots (MRs). Path planning is the calculation of a feasible path from a start node to a goal node in a map or grid without colliding with obstacles on the way [7]. It requires MR to be equipped with sensors, onboard computers, and motion systems to plan and move in partially or completely unknown environments.

Path planning refers to the problem of finding a route (path) between a start node and a goal node on a map (grid) [8]. Many well-established algorithms have been proposed to solve the path-planning problems for MR. The former approach includes search-based planning algorithms: Dijkstra [9], *A*^*∗*^ [10], *D*^*∗*^ [11], etc. Due to their universality and ease of implementation, these algorithms achieve significant results in searching for paths, but the search time exponentially grows with the resolution size and search depth of the map; sampling-based planning algorithms: rapidly random-exploring tree (RRT) [12], probabilistic roadmap (PRM) [13], etc. The advantage of these algorithms is that they are effective and fast in high-dimensional path search, the disadvantage is that these algorithms usually sample the environment for random search to find paths, the results are often not optimal, and it is difficult to find a feasible path in environments with narrow passages. Artificial potential field (APF) [14] and BUG algorithm [15] are widely used in path planning for local obstacle avoidance. Although these algorithms are computationally simple for dynamic obstacle environments and fast in path search, the optimal path is often not obtained, and the search path may be erroneous when the obstacles are large in a complex environment. Another type of algorithm is the intelligent algorithm: it is an algorithm that people model by nature-inspired or human mind to imitate solving problems [16]. Typical algorithms are particle swarm optimization (PSO) [17], ant colony optimization (ACO) [18], neural network (NN) [19], and other algorithms. Intelligent algorithms play an effective role in solving complex dynamic environments, but there are common problems such as slow computation speed, poor stability, poor real-time performance, and easy to fall into local optimality.

All of the abovementioned algorithms have their own advantages and disadvantages. Most of the studies treat MR as a mass point to simplify the path-planning problem and ignore dynamic uncertainties during motion [20], resulting in a searched path too close to the obstacle.

### 1.1. Related Work

Recently, due to the development of artificial intelligence, Q-learning, the most commonly used algorithm in reinforcement learning, has been increasingly used in the field of path planning. Reinforcement learning is a class of unsupervised algorithms [21] that emphasize the learning process of the MR interacting with its environment. The goal of the MR is to evaluate the action to be chosen in each state of its environment by learning a state-action value function for that state.

A common approach is to improve the initialization of Q-learning by combining it with other algorithms. In [22, 23], algorithms improved the initialization of Q-learning with a bionic algorithm [23] to accelerate the learning speed and significantly reduce the computation time of the mobile robot; to solve the problem of excessive dimensionality of Q-table, the studies [24–26] combine neural network with Q-learning, which converge to the optimal policy faster and reach the goal point with less distance. Zhang et al. [27] proposed a self-adaptive reinforcement-exploration Q-learning (SARE-Q) to address the problems of many repetitions and uneven exploration of classical Q-learning. Simulation experiments show that the algorithm has significant advantages over classical Q-leaning in terms of the average number of turns, success rate, and the number of shortest planned routes. In order to solve the problems of slow convergence speed and long planning paths when robots use the Q-learning algorithm to plan paths in unknown environments, Zhao et al. [28] proposed the experience-memory Q-learning (EMQL) algorithm, which improves the autonomous learning capability of mobile robots by continuously updating the shortest distance from the current state node to the starting point. The comparison results from the planning time, the number of iterations, and path length show that the algorithm has obvious advantages in terms of convergence speed and optimization capability.

To overcome the above limitations, this study proposes an improved Q-learning [29] to solve the path-planning problem of MR and refers to the proposed algorithm as the potential and dynamic Q-learning (PDQL). The proposed algorithm exploits the superiorities and minimizes the limitations of Q-learning in path planning for MR. The proposed approach enables the MR to escape from dead-end paths blocked by obstacles. The effectiveness, superiority, and rapidity of the PDQL algorithm are demonstrated through simulation and comparison experiments.

### 1.2. Original Contributions

The PDQL algorithm proposed in this study is applied to solve the path planning for MRs in an unknown environment with the following main contributions:

1. The PDQL is a novel proposal that can find the feasible path for MR in an unknown environment, outperforming motion-planning proposals based on the state-of-the-art path-planning algorithms.
2. This study hybridizes Q-learning with APF to initialize Q-table, avoiding the random movement around the start point at the beginning of the algorithm.
3. The proposed algorithm changes the constant reward into a dynamic reward, speeds up the convergence of the algorithm, and avoids the algorithm from falling into a dead-end path blocked by obstacles.
4. By comparing with other algorithms, it is proved that the proposed algorithm in this study can solve the MR path-planning problems in an unknown environment considering the path length, collision avoidance, and smoothness.

The MR path planning is a broad research area involving planning, obstacle avoidance, and algorithms. To better describe the problem and prove the effectiveness of the proposed PDQL algorithm, the remainder of this study is organized as follows: first, the path-planning problem is described in Section 2; then the proposed PDQL algorithm is described in Section 3; the comparative experimental and simulation results *f* are presented in Section 4; and finally, conclusions are drawn and future work is discussed in Section 5.

## 2. Preliminaries

In this section, this study first describes the MR path-planning problem and then outlines the classical Q-learning.

### 2.1. Problem Formulation

The task studied in this study is the path-planning problem of a mobile robot in an unknown environment. The information in the environment (obstacle location, shape, and orientation) is completely unknown before the mobile robot performs a path-planning task, and only the starting and goal points are known. Mobile robots need to plan the shortest path length from the starting point to the goal point without colliding with obstacles in the environment [30].

This study studies path planning on an eight-way connected two-dimensional grid of nodes. The path-planning problem can be defined in the following forms: *S* ∈ *ℝ*^*n*^ for state space (MR workspace); *S*_*obs*_ for obstacle space (MR unreachable space); *γ* for free space (MR reachable space); *s*_start_(*s*_start_ ∈ *S*_free_) for initial state (MR starting position); and *s*_goal_(*s*_goal_ ∈ *S*_free_) for goal state (MR goal position). The purpose of MR is to calculate a path *N*(*N*=[*s*_start_, *s*_1_, *s*_2_,…, *s*_goal_] ∈ *S*_free_) that does not collide with obstacles.

The MR moves from one grid *s*_a_ to one of the eight adjacent grids *s*_b_, which is defined as a cost function as cos  *t*, as shown in equation (1).

(1) $$\begin{array}{c} \cos\;t=\left\{\begin{array}{cc} 1, & s_{a}\text{ to }s_{b}\;\text{hor.or ver, }\\ \sqrt{2}, & s_{a}\text{ to }s_{b}\;\text{diagonal},\\ \infty , & s_{b}\in S_{obs}. \end{array}\right) \end{array}$$

The problem studied in this study is to plan a path that does not collide with obstacles so that the MR reaches the end point from the starting point with a shorter path length.

**Figure 1:**
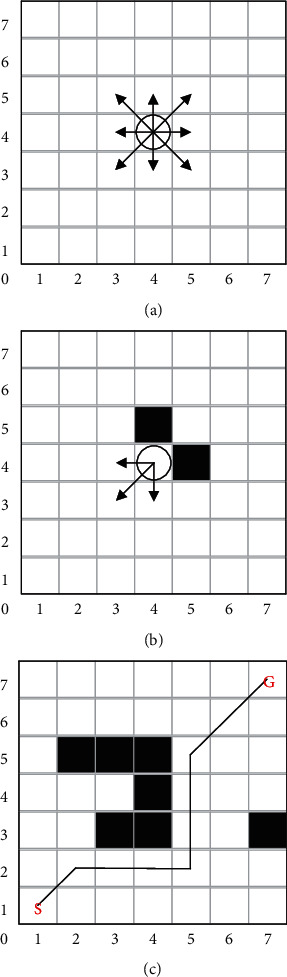
Path-planning problem formulation.

To illustrate the planning problem using the PDQL in this study, the path-planning process is shown in Figure 1. The MR is defined as a circle of radius *r*, and the working environment of MR is divided into a grid map with sides of 7*∗*2*r*. In a Cartesian coordinate, the MR aims to plan a feasible path from the start *S* (1, 1) to the goal *G* (7, 7) without collision with obstacles. For better obstacle avoidance, this study defines (Figure 1(a)) the following: when there are no obstacles around, the MR can move to the adjacent eight directions (north, northeast, east, southeast, south, southwest, west, and northwest). The MR cannot diagonally cross the obstacles' barrier. When the obstacles are located around the MR, the movement directions of the MR are shown in Figure 1(b). Figure 1(c) shows the path planned by the proposed PDQL algorithm.

### 2.2. Q-Learning Algorithm

**Figure 2:**
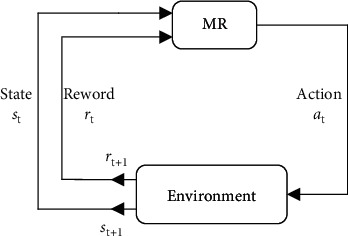
Framework of reinforcement learning.

Q-learning [29] is a value-based reinforcement learning algorithm proposed by Watkins in 1989. The MR path planning can be expressed as follows: at each discrete time series (offline strategic temporal difference) [31]. The framework of the reinforcement learning is shown in Figure 2.

The MR can receive a state *s*_*t*_ ∈ States (coordinates of the MR in the current state) from the environment and interact with the environment through action *a*_*t*_ ∈ Actions (direction and distance of MR movement). The environment will provide a new state *s*_*t*+1_ ∈ States (coordinates of the MR in the next state) and also give an immediate return *r*_*t*+1_, where States is the set of states in the maritime environment, and Actions is the set of actions available at the state *s*_*t*_. The MR interacts with the environment through continuous feedback, generating more data (states and returns) and using the new data to further improve its own behavior. Q-table is the expectation that *Q*(*s*_*t*_, *a*_*t*_) can gain by taking action *a*_*t*_ in state *s*_*t*_, which is updated by the equation (2).

(2) $$\begin{array}{c} Q\left(s_{t},a_{t}\right)\leftarrow \left(1-\alpha \right)*Q\left(s_{t},a_{t}\right)+\alpha *\left[r_{t+1}+\gamma \underset{a}{\max}Q\left(s_{t+1},a_{t+1}\right)\right]. \end{array}$$

The above function can also be written as equations (3) and (4):

(3) $$\begin{array}{c} Q\left(s_{t},a_{t}\right)\leftarrow \left(1-\alpha \right)*Q\left(s_{t},a_{t}\right)+\alpha *\left[r_{t+1}+\gamma V\left(s_{t+1}\right)\right], \end{array}$$

(4) $$\begin{array}{c} V\left(s_{t}\right)\leftarrow V\left(s_{t}\right)+\alpha *\left[r_{t+1}+\gamma V\left(s_{t+1}\right)-V\left(s_{t}\right)\right], \end{array}$$

where *α* (0 ≤ *α* ≤ 1) is a learning rate parameter, and *γ* (0 ≤ *γ* ≤ 1) is a discount rate parameter.

After the convergence of the Q-learning, the mobile robot can obtain a convergent Q-table to guide it on how to obtain the maximum cumulative reward and eventually learn the optimal action (optimal strategy) to complete the path-planning task. Watkins et al. [29] show that when the states and actions are finite, the Q-values can be represented as a Q-table. Q-learning converges to the optimal policy under the condition that the mobile robot always selects an action after convergence when each state and action has an infinite number of visits to the Q-table, and the maximum Q-values for each state are obtained as shown in equation (5)as follows:

(5) $$\begin{array}{c} V^{*}\left(s_{t}\right)=\underset{a}{\max}Q\left(s_{t},a_{t}\right). \end{array}$$

The pseudocode of the classical Q-learning algorithm is summarized as in Algorithm 1.

In this study, the Q-learning is applied to the MR path planning for the following reasons:

1. Reinforcement learning has good interaction with the environment without the need for positive or negative labels [32]. The MR gains current knowledge by exploring and learning from the environment, and improves their operational strategies to adapt to the environment.
2. The Q-learning algorithm is highly exploratory and is an iterative trial-and-error process, where multiple attempts are made for each possible pair of state actions to obtain the optimal policy as long as time allows.
3. Q-learning uses off-policy [33], and the selection of actions according to the target strategy can be used to control the distance between the MR and the obstacle.

Although Q-learning has many advantages, there are several shortcomings as follows:

1. In the initial stage, Q-table is initialized to zero or normal-distributed numbers, the motion of MR is completely random and easy to collide with obstacles, resulting in the loss of MR, and the convergence speed is slow and time-consuming.
2. Q-learning requires a certain memory to store the tracking Q-table. When MR has *m* states and *n* actions, the dimension of the constituted Q table is *m∗n*, and by choosing the maximum Q value to determine the next move's direction, a total of *m∗*(*n* − 1) times need to be compared, with the more complex state space and actions, which will exponentially increase the amount of computation, resulting in long computation time [30].
3. It will lead to local optimum when the environment is complex, and it is easy to fall into a dead-end path blocked by the obstacles.

According to the superior characteristics of Q-learning and avoiding its shortcomings, this study proposes a novel algorithm PDQL based on Q-learning to solve the path-planning problem for MR in an unknown environment. To provide a prior knowledge for MR, this algorithm combines APF with Q-learning to initialize Q-table; the dynamic reward is set to speed up the convergence of the Q-learning. The path length, computing time, and turning angle are improved compared to classical Q-learning.

## 3. The Proposed PDQL Algorithm

In this section, the proposed PDQL algorithm is elaborated in five stages for solving the path-planning problem for MR in an unknown environment. To better describe the studied content, the MR path-planning problem discussed in this study has the following premises:

1. The MR and the obstacle environment are three-dimensional objects in practice. To simplify the problem, this study ignores the height of the MR and obstacles and treats them as objects in two dimensions.
2. The location, shape, and size of obstacles in the environment are unknown to MR, and the MR only knows the start and goal positions.
3. The task of the MR is to reach the target from the start with the shortest possible path length.

### 3.1. Q-Table Initialization

The Q-learning algorithm usually initializes the Q-table as zeros or normally distributed random numbers, and the absence of prior knowledge of the environment leads the MR to randomly choose actions in the exploration phase, resulting in slow convergence and long computation time of the algorithm. To optimize this problem, this study uses the APF combined with the Q-learning algorithm to optimize the initial Q-table for path planning. The reasons are as follows: it is easy to implement in grid maps, provides prior knowledge of the environment for MR, and speeds up the computation and convergence.

The information in the environment (obstacle shape, size, and location coordinates) is unknown for MR, so Coulomb's law is used to model the APF for the grid environment. The repulsive force from the obstacle is not calculated, and only the gravitational force is generated by the starting point. The equations are shown in equations (6) and (7)as follows:

(6) $$\begin{array}{c} U_{a}\left(s_{t}\right)=\frac{1}{2}k_{a}\rho_{g}^{2}\left(s_{t}\right), \end{array}$$

(7) $$\begin{array}{c} U_{n}\left(s_{t}\right)=U_{a}\left(s_{t}\right), \end{array}$$

where *U*_*a*_(*s*_*t*_) is the gravitational field producing gravitational force in state *s*_*t*_; *U*_*n*_(*s*_*t*_) is the total potential energy of state *s*_*t*_; *ρ*_*g*_(*s*_*t*_) is the Euclidean distance between state *s*_*t*_ and the center of the target point; and *k*_a_ is the scale factor.

The grid of the MR workspace is modeled according to the above method, and in order to make the potential energy range in space between (0, 1) and the highest potential energy at the goal point and the lowest potential energy in the obstacle region, the vector field is normalized using equation (8).

(8) $$\begin{array}{c} U\left(s_{t}\right)=\left|\frac{U_{\max}-U_{n}\left(s_{t}\right)}{U_{\max}}\right|, \end{array}$$

where *U*(*s*_*t*_) is the potential energy in state *s*_*t*_; *U*_max_ is the highest potential energy in state *s*_t_; and through equation (8), a potential energy field is constructed for each grid in the known environment, with a grid potential energy of 1 at the target point and a potential energy of 0 at the obstacle grid, forming a monotonically increasing potential energy field from the starting point to the target point.

The *Q*-table is initialized using equation (8) as shown in equation (9).

(9) $$\begin{array}{c} Q_{0}\left(s_{0},a_{0}\right)=U\left(s_{t}\right). \end{array}$$

By applying APF to Q-table initialization, the MR is provided with a prior knowledge of the known environment, which avoids the disadvantage of slow convergence caused by the random motion in the exploration phase. This section lays the foundation for defining the action and reward function in the next section.

### 3.2. Action Selection

In the grid map, the MR uses continuous learning based on the environmental information, and gets rewarded and punished by interacting with the environment. The MR gets rewarded when moving to the white grid, penalized when reaching the color grid, and converged to the optimal value by updating the formula through Q-learning. To avoid obstacles and reach the target point with the shortest path length, eight directions of movement are defined for the MR (north, northeast, east, southeast, south, southwest, west, and northwest). When the radius of MR is defined as *r*=1m, the corresponding movement and movement distance are as follows:

  action 1: *a*_1_=move north 2m;
  action 2: $a_{2}=\text{move northeast }2\sqrt{2}\text{m;}$
  action 3: *a*_3_=move east 2m;
  action 4: $a_{4}=\text{move southeast }2\sqrt{2}\text{m;}$
  action 5: *a*_5_=move south 2m;
  action 6: $a_{6}=\text{move southwest }2\sqrt{2}\text{m;}$
  action 7: *a*_7_=move west 2m;
  action 8: $a_{8}=\text{move northwest }2\sqrt{2}\text{m;}$

### 3.3. Reward Function

The reward function is used to determine the value of the behavior, and the MR interacts with the environment according to the reward function to adjust the action strategy by the reward value [24]. The right reward function helps to reinforce the desired behavior and punish the improper behavior. In previous reinforcement-learning path planning, the reward value is usually a static constant, which leads to its random search in the environment, resulting in increased convergence time. To solve this problem, a dynamic reward function is proposed in the PDQL algorithm, which provides the goal point and the current position as prior knowledge to the MR. When the MR is closer to the goal point, the larger the reward obtained, prompting it to move in the direction of the goal point and speeding up the convergence. To reduce the computation, this study uses Manhattan distance for calculation.

The reward functions are shown from equations (10) to (14).

(10) $$\begin{array}{c} r=r_{s}*\left(1+r_{d}\right), \end{array}$$

(11) $$\begin{array}{c} r_{s}=\left\{\begin{array}{c} 1,\text{ hor. or ver. movement,}\\ 2,\;s_{t+1}\text{ is the start node,}\\ 1/\sqrt{2},\text{ diagonal movement,}\\ 10,\;s_{t+1}\text{ is the target node,}\\ -\inf,\;s_{t+1}\text{ is the for bidden node,} \end{array}\right. \end{array}$$

(12) dt=y−targetyt+x−targetxt,

(13) dt+1=y−targetyt+1+x−targetxt+1,

(14) $$\begin{array}{c} r_{d}=\frac{d_{t}-d_{t+1}}{\left|d_{t}-d_{t+1}\right|}, \end{array}$$

where *r*_s_ is the static reward; *r*_*d*_ is the dynamic reward; *d*_*t*_ is the Manhattan distance from the target point in the state *s*_*t*_; *d*_*t*+1_ is the Manhattan distance from the target point in the next state *s*_*t*+1_; (*x*_*t*_, *y*_*t*_) is the coordinate in the state *s*_*t*_; (*x*_*t*+1_, *y*_*t*+1_) is the coordinate in the state *s*_*t*+1_; and (*x*_target_, *y*target) is the coordinate in the goal point.

### 3.4. Convergence for PDQL

To demonstrate the convergence of the proposed algorithm applied to the MR path-planning task, assuming that *Q*_*n*_′(*s*_*t*_, *a*_*t*_) is the estimate of *Q*(*s*_*t*_, *a*_*t*_) for the nth update of the Q-value, the error of *Q*_*n*_′(*s*_*t*_, *a*_*t*_) is defined as shown in equation (15).

(15) $$\begin{array}{c} \delta_{n}=\left|Q_{n}^{\prime }\left(s_{t},a_{t}\right)-Q\left(s_{t},a_{t}\right)\right|. \end{array}$$

After *n* + 1th update of the *Q*-value, the error of *Q*_*n*+1_′(*s*_*t*_, *a*_*t*_) is shown as in equation (16).

(16) $$\begin{array}{c} \delta_{n+1}=\left|Q_{n+1}^{'}\left(s_{t},a_{t}\right)-Q\left(s_{t},a_{t}\right)\right|\leq \left|\left(1-\alpha \right)\left(Q_{n}^{\prime }\left(s_{t},a_{t}\right)-Q\left(s_{t},a_{t}\right)\right)\right|+\alpha \gamma \underset{a}{\max}\left|Q_{n}^{\prime }\left(s_{t+1},a_{t+1}\right)-Q\left(s_{t+1},a_{t+1}\right)\right|\leq \left(1-\alpha \right)\delta_{n}+\alpha \gamma \delta_{n}<\delta_{n}. \end{array}$$

From the proof of equation (16), the (*n* + 1)th error *δ*_*n*+1_ is smaller than the nth error *δ*_*n*_. As the PDQL algorithm iterates, the Q-value converges to a definite value. The MR will move from the starting point to the goal point with the optimal action strategy.

### 3.5. PDQL for Path Planning

In this study, the proposed PDQL algorithm is applied to the path-planning problem of MR in an unknown environment, and its basic ideas are as follows: the APF is used to initialize Q-table, to provide the prior knowledge in the environment to the MR. The dynamic reward is combined to optimize the reward function, to induce the MR to move toward the goal point, and to control the distance between MR and obstacles.

## 4. Simulation Results

It is difficult to directly apply the PDQL algorithm to the path-planning problem of MR, and it requires repeated training to obtain the optimal action strategy, therefore, this study demonstrates the performance and generality of the PDQL through several numerical simulations in this section.

### 4.1. Environments

In this study, the environment of the MR working is simplified by using a grid map. A circle of radius is used to represent the MR, a square of side length is used to represent each grid node in the working space, white grid indicates the feasible area, and black grid indicates the obstacle (the area where the MR is forbidden to reach). The center of each grid is marked by a Cartesian coordinate, with the *x* axis indicating the horizontal direction and the *y* axis indicating the vertical direction. Therefore, the first and second dimensions of the grid map represent the *x* coordinate and *y* coordinate of the grid in the map, respectively.

**Figure 3:**
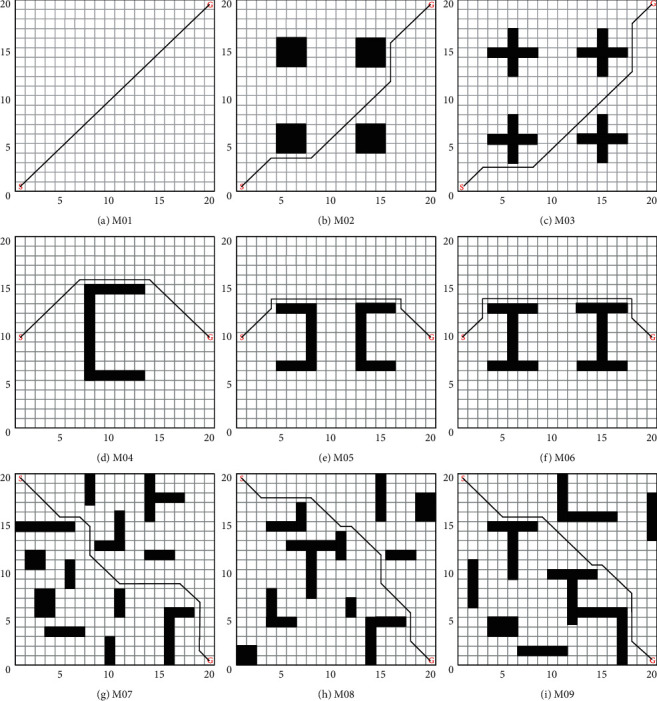
Path planning (solution) for MR in different test environments (each map shows the best path obtained by the PDQL algorithm, *S* is the starting point, and *G* is the goal point). (a) M01. (b) M02. (c) M03. (d) M04. (e) M05. (f) M06. (g) M07. (h) M08. (i) M09.

The simulation maps are shown in Figure 3 from M01 to M09, simulating the MR in different environments with locations. From simple to complex environments, numerous challenges in the field of path planning are covered: problems such as planning the short path over long distances and trap points due to local minima.

### 4.2. Performance Metrics

To test the effectiveness, safety, and speed of the proposed PDQL algorithm in a comprehensive and concrete way, the MR path is evaluated in three performance metrics: path length equation (17), turning angle equation (21), and computing time.

#### 4.2.1. Path Length (m)

The path length is an important indicator to test the path performance. When the MR tracks the path, the shorter the distance traveled, the shorter time required to complete the task, and the less energy consumed. Therefore, this study defines the path length of the MR from the starting position to the goal position as shown in equation (17).

(17) $$\begin{array}{c} \text{Path Length}\left(m\right)=\sum_{i=0}^{n}\sqrt{{\left(y_{i+1}-y_{i}\right)}^{2}+{\left(x_{i+1}-x_{i}\right)}^{2}}\text{ ,} \end{array}$$

where *i*=0,1,2, ..., *n*, when *i*=0, the MR is at the starting position *S*=(*x*_*o*_, *y*_*o*_), when *i*=n, the MR is at the target position *T*=(*x*_*n*_, *y*_*n*_), (*x*_*i*_, *y*_*i*_) represents the coordinates of the current state of the MR, and (*x*_*i*+1_, *y*_*i*+1_) represents the coordinates of the next state of the MR.

#### 4.2.2. Turning Angle (rad)

The turning angle is the sum of the change in the heading angle of MR from the start to the goal. When the turning angle is smaller, the path is smoother, less energy is consumed, and the completion time of the mission is shorter. This study defines the turning angle of MR from the start to the goal position as shown in equations (18)∼(21).

(18) ai=y−iyi−12+x−ixi−12,

(19) bi=y−i+1yi2+x−i+1xi2,

(20) $$\begin{array}{c} c_{i}=\sqrt{{\left(y_{i+1}-y_{i+1}\right)}^{2}+{\left(x_{i+1}-x_{i+1}\right)}^{2}}, \end{array}$$

(21) $$\begin{array}{c} \text{Angle}\left(\text{rad}\right)=\sum_{i=1}^{n}\left(\pi -\mathrm{arc}\;\cos\frac{a_{i}^{2}+b_{i}^{2}-c_{i}^{2}}{2*a_{i}*b_{i}}\right), \end{array}$$

where *i*=0,1,2, ..., *n*, (*x*_*i*_, *y*_*i*_) represents the coordinates of the current state of MR; (*x*_*i*+1_, *y*_*i*+1_) represents the coordinates of the next state of MR.

#### 4.2.3. Turning Angle (rad)

This measure corresponds to the time consumed by the algorithm through iterative computation.

Functions of the path length, turning angle, and computation time to evaluate the path can be used to fully evaluate the degree of excellence of the algorithm to compute the path. The smaller the path length, angle, and computation time, the better is the proposed algorithm.

### 4.3. Parameters Selection

Before the PDQL algorithm is executed, the values of the two parameters: learning rate *α* (0 ≤ *α* ≤ 1) and decay rate *γ* (0 ≤ *γ* ≤ 1) in the Q-value update equation, need to be determined based on the evaluation function of Section 4. According to Watkins et al. [29], when the *α* value is small, the agent goes through all states in the environment and calculates all possible actions, and the *Q* value converges to the optimal value. When the *γ* value is large, it can expand the exploration range of the agent and prevent the agent from falling into the problem of local optimum. Therefore, based on the above theory, when *γ*=0.9, this study records the number of iterations that path length converges to the optimum. The test is repeated 30 times to take the average value; considering the computing time of MR, both *α* and *γ* are taken as 0.9.

To demonstrate the effectiveness and generalizability of the proposed PDQL algorithm in local path planning, the PDQL algorithm is analyzed and compared with the commonly used MR local path-planning algorithms [7]: RRT, APF, and fuzzy [34]. The parameters are set as shown in Table 1.

Max is the maximum number of iterations. For RRT, *p* is the random adoption probability, *size* is the step length, and Max_attempt is the maximum number of sampling. For APF, *ρ*_o_ is the obstacle influence factor, and *k*_a_ and *k*_*r*_ are the scale factors. For Fuzzy, Max Turn is the maximum turning angle for each time.

### 4.4. PDQL for MR Path Planning

In this section, the proposed PDQL algorithm enables the MR to perform the task with the optimal path length from the starting point to the goal point. To verify the effectiveness and generalizability of the proposed algorithm, tests were conducted on the nine grid maps in Figure 3. Each map shows the best path obtained by the PDQL algorithm (black solid line), *S* is the starting point, and *G* is the goal point.

The optimal (suboptimal or optimal under optimal conditions) results of PDQL in 30 repeated tests (the path with the smaller turning angle and computing time under the same path-length condition is taken) are shown in Figure 3 (the paths of other compared algorithms are not indicated in Figure 3 for a clearer representation of the paths of PDQL). Experimental data and analysis results are recorded in Table 2, and it is checked whether there is a significant difference between the PDQL algorithm and other comparison algorithms by *t*-test (considering a 0.05 significance level). Among the results, the path length and turning angle of APF and fuzzy are the same in each experimental result, and there is no *t*-test.

**Figure 4:**
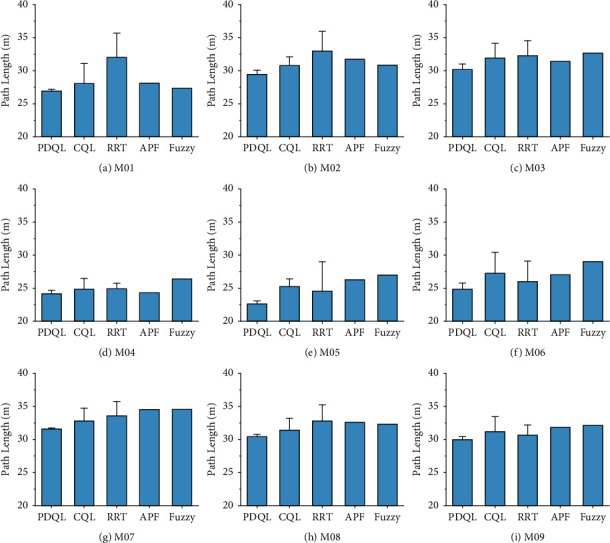
Compared mean path length of various path-planning algorithms for MR (the interval on each bar denotes the standard deviation of the path length). (a) M01. (b) M02. (c) M03. (d) M04. (e) M05. (f) M06. (g) M07. (h) M08. (i) M09.

**Figure 5:**
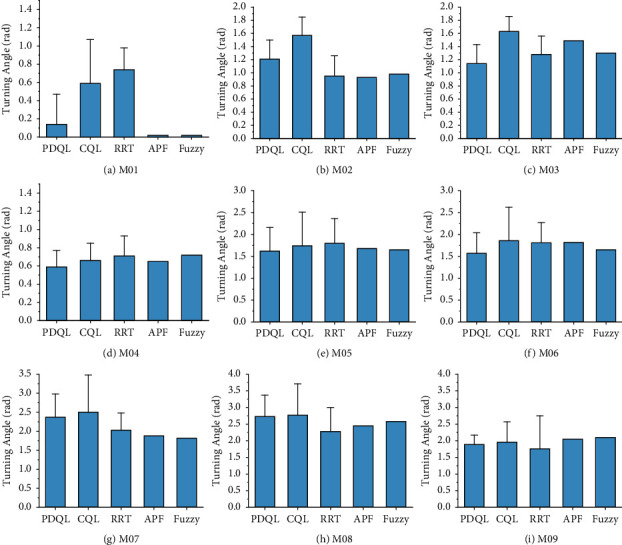
Compared mean turning angle of various path-planning algorithms for MR (the interval on each bar denotes the standard deviation of the turning angle). (a) M01. (b) M02. (c) M03. (d) M04. (e) M05. (f) M06. (g) M07. (h) M08. (i) M09.

**Figure 6:**
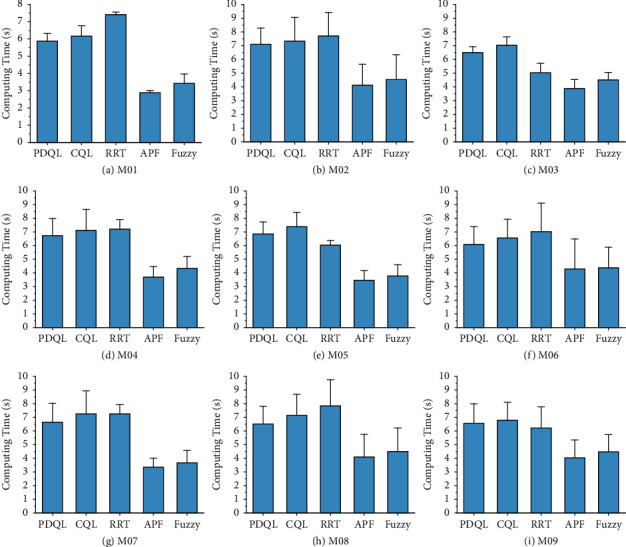
Compared mean computing time of various path-planning algorithms for MR (the interval on each bar denotes the standard deviation of the computing time). (a) M01. (b) M02. (c) M03. (d) M04. (e) M05. (f) M06. (g) M07. (h) M08. (i) M09.

Compared mean path length, turning angle, and computing time of various path-planning algorithms for MR are shown in Figures 4–6 (the interval on each bar denotes the standard deviation).

It can be seen that in the nine test maps, the PDQL can effectively solve the path-planning problem of MR from the starting position to the goal position without collision with obstacles under short path-length condition, and it can achieve excellent results in each environment. These results show that the proposed approach achieves the optimal path length in different environments, which demonstrates its practicality, generalizability, and robustness. Compared to the existing methods, the proposed approach can effectively find the optimal local path. The limitation is that the computing time is longer compared to the comparison algorithms, and it will exponentially grow with the size of the map. The proposed approach has the smallest standard deviation among the comparison algorithms in all cases, which demonstrates the superiority and stability of the PDQL algorithm under different environments. In the experimental process, the randomness of the path length and turning angle derived by the RRT algorithm is too large (standard deviation) and is not suitable for the MR path planning. For APF and fuzzy, although the stability of the path length and turning angle derived each time is good, it requires adjusting the corresponding parameters to avoid the problem of falling into local minima. The APF is not able to calculate the results when the obstacles are too dense.

These results of global path planning for MR show that our method achieves the optimal path length and turning angle in different environments, which demonstrates its practicality, generalizability, and robustness. Compared to the existing methods, the proposed approach can effectively escape from trap points due to local minima and find the optimal global path. The limitation is that the computing time is longer compared to the comparison algorithms, but it is within the acceptable range.

## 5. Conclusions

In this study, the PDQL for MR path-planning algorithm is proposed, and simulation experiments are conducted in different environments. The environment in the path planning is unknown, and the PDQL can effectively solve the MR path-planning task. By combining APF with the Q-learning algorithm to initialize the Q-table and setting the static and dynamic reward functions to provide the prior knowledge in the environment to the MR, the convergence speed and computing time of CQL are accelerated.

To demonstrate the effectiveness and generality of the proposed algorithm, simulation experiments are set up, fully considering the unknown environment in the actual work. It demonstrates that the proposed PDQL algorithm can effectively solve the path-planning task, and the mean, standard deviation, and *t*-test of the data analysis show that the proposed algorithm yields the smallest path length and smoothness, which significantly speeds up the computation time and convergence speed of the CQL. The problem of MR falling into local minima and not being able to derive effective paths is avoided.

In future work, the direction of the work can be derived from the problem that Q-learning computation time exponentially grows with the range of the environment. To solve the problem of excessive dimensionality of Q-tables in reinforcement learning, Q-learning is combined with neural networks in order to effectively reduce the computation time and the waiting time for MR to perform actions [24, 25], and on the other hand, for the multi-MR path-planning problem [35]. Q-learning minimizes the path length and arrival time of all robots in the environment to their respective destinations and reduces the turning angle of each robot.

## Figures and Tables

**Algorithm 1:**
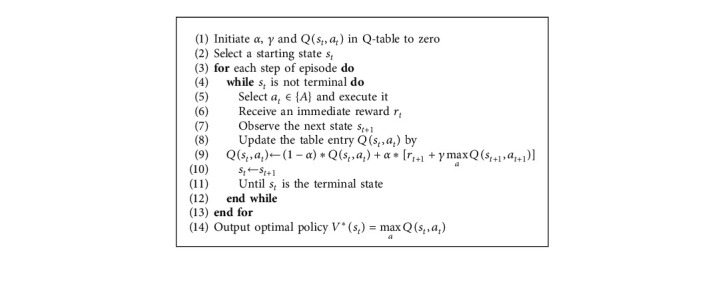
Classical Q-learning pseudocode.

**Table 1:** Parameter setting of PDQL, CQL, RRT, APF, and fuzzy.

Algorithms	Parameter selection	Max
DMQL	*α*=0.9, *γ*=0.9, *ρ*_*o*_=2, *k*_*a*_=1.5, *k*_*r*_=1.5	100
CQL	*α*=0.9, *γ*=0.9	100
RRT	*p*=0.5, size=20, attempt=10000	
APF	*ρ*_o_=2.5, *k*_a_=2, *k*_*r*_=2	—
Fuzzy	Max Turn=*π*/3	

**Table 2:** A comparison between PDQL, CQL, RRT, APF, and fuzzy for path planning. Both the lowest (best) mean and the values of path length bigger than the level 0.05 of significance are highlighted.

Env	Statistics	PDQL	CQL	RRT	APF	Fuzzy
M01	Path length	**26.93**	28.06	32.02	27.36	28.12
	*t*-test	—	4.94*e* − 2	2.16*e* − 8	—	—
	Angle	0.14	0.59	0.74	0.02	0.02
	Time	5.88	6.17	7.41	2.89	3.43

M02	Path length	**29.45**	30.79	32.98	31.75	30.83
	*t*-test	—	8.06*e* − 6	3.57*e* − 7	—	—
	Angle	1.21	1.57	0.95	0.93	0.98
	Time	7.11	7.33	7.71	4.12	4.54

M03	Path length	**30.17**	31.91	32.26	31.42	32.68
	*t*-test	—	2.84*e* − 4	3.56*e* − 5	8.29*e* − 4	1.69*e* − 6
	Angle	1.14	1.63	1.28	1.49	1.30
	Time	6.50	7.03	5.03	3.88	4.51

M04	Path length	**24.16**	24.85	24.91	24.30	26.40
	*t*-test	—	3.47*e* − 2	1.32*e* − 4	—	—
	Angle	0.59	0.66	0.71	0.65	0.72
	Time	6.73	7.12	7.21	3.69	4.32

M05	Path length	**22.63**	25.23	24.53	26.28	26.99
	*t*-test	—	0.27*e* − 2	2.77*e* − 2	—	—
	Angle	1.62	1.74	1.80	1.68	1.65
	Time	6.85	7.38	6.03	3.46	3.78

M06	Path length	**24.83**	27.26	25.99	27.02	29.02
	*t*-test	—	2.94*e* − 4	**5.71*e*** − **2**	—	—
	Angle	1.57	1.86	1.81	1.82	1.65
	Time	6.08	6.56	7.02	4.29	4.37

M07	Path length	**31.60**	32.81	33.57	34.55	34.56
	*t*-test	—	1.94*e* − 3	3.01*e* − 5	—	—
	Angle	2.37	2.50	2.03	1.88	1.82
	Time	6.64	7.26	7.26	3.35	3.67

M08	Path length	**30.44**	31.41	32.81	32.61	32.31
	*t*-test	—	6.61*e* − 3	9.84*e* − 6	—	—
	Angle	2.73	2.77	2.28	2.45	2.58
	Time	6.51	7.14	7.84	4.10	4.50

M09	Path length	**29.99**	31.18	30.67	31.83	32.15
	*t*-test	—	8.54*e* − 3	2.57*e* − 2	—	—
	Angle	1.89	1.96	1.76	2.05	2.10
	Time	6.56	6.79	6.22	4.04	4.48

## Data Availability

The data used to support the findings of this study are available from the corresponding author.
